# Airway symptoms and lung function in the local population after the oil tank explosion in Gulen, Norway

**DOI:** 10.1186/1471-2466-12-76

**Published:** 2012-12-12

**Authors:** Jens-Tore Granslo, Magne Bråtveit, Bjørg Eli Hollund, Ågot Irgens, Cecilie Svanes, Nils Magerøy, Bente Elisabeth Moen

**Affiliations:** 1 Department of Occupational Medicine, Haukeland University Hospital, Bergen, Norway; 2 Department of Public Health and Primary Health Care, University of Bergen, Bergen, Norway; 3 Institute of Medicine, University of Bergen, Bergen, Norway

**Keywords:** Airway symptoms, Environmental pollutants, Explosion, Lung function

## Abstract

**Background:**

Oil tanks containing a mixture of hydrocarbons, including sulphuric compounds, exploded and caught fire in an industrial harbour. This study assesses airway symptoms and lung function in the nearby population 1½ years after the explosion.

**Methods:**

A cross-sectional study included individuals ≥18 years old. Individuals living <6 km (sub-groups <3km and 3–6 km) from the accident site formed the exposed group, individuals living >20 km away formed a control group. A questionnaire and spirometry tests were completed by 223 exposed individuals (response rate men 70%, women 75%) and 179 control individuals (response rate men 51%, women 65%). Regression analyses included adjustment for smoking, occupational exposure, atopy, infection in the preceding month and age. Analyses of symptoms were also adjusted for stress reactions related to the accident.

**Results:**

Exposed individuals experienced significantly more blocked nose (odds ratio 1.7 [95% confidence interval 1.0, 2.8]), rhinorrhoea (1.6 [1.1, 3.3]), nose irritation (3.4 [2.0, 5.9]), sore throat (3.1 [1.8, 5.5]), morning cough (3.5 [2.0, 5.5]), daily cough (2.2 [1.4, 3.7]), cough >3 months a year (2.9 [1.5, 5.3]) and cough with phlegm (1.9 [1.2, 3.1]) than control individuals. A significantly increasing trend was found for nose symptoms and cough, depending on the proximity of home address to explosion site (daily cough, 3-6km 1.8 [1.0, 3.1], <3km 3.0 [1.7, 6.4]). Lung function measurements were significantly lower in the exposed group than in the control group, FEV_1_ adjusted mean difference −123 mL [95% confidence interval −232, -14]), FEV_1_% predicted −2.5 [−5.5, 0.5], FVC −173 mL [− 297, -50], FVC% predicted −3.1 [− 5.9, -0.4], and airway obstruction (GOLD II/III).

**Conclusions:**

Based on cross sectional analyses, individuals living in an area with air pollution from an oil tank explosion had more airway symptoms and lower lung function than a control group 1½ years after the incident.

## Background

One morning in May 2007, an oil tank exploded, caught fire and ignited a nearby tank with similar contents. Sooty smoke spread in a north-easterly direction until the fire was extinguished in the afternoon. The oil tanks were located in a one-square-kilometre industrial harbour area by a fiord in Gulen municipality, Western Norway. The industrial harbour is located in a rural area. Except for the industrial harbour there are no industrial emission sites and the nearest town is more than 50 kilometres air distance to the south.

No one died or was seriously injured, but for many months the local population reported an unpleasant smell from the area. In a report to the authorities, local physicians described various health symptoms in the population, such as sore throats and headaches [1]. The event was discussed in the mass media for a long time. Health concerns called for an initiative and, in 2008, the national health authorities decided to carry out a health examination of the local population.

Few studies have examined persistent respiratory effects after chemical disasters or other air-polluting accidents. Three months after the catastrophe at the World Trade Center (WTC) in 2001, 191 federal office employees (response rate 68%) who worked in three federal buildings near the WTC and a control group of 155 (response rate 47%) federal office employees in Dallas, Texas, participated in a cross-sectional questionnaire based survey. The prevalence of upper and lower airway symptoms and also of mental health symptoms was higher among the NY employees [2]. About one year (8–16 months) after the WTC accident a self-administrated questionnaire based study was conducted on 2520 residents from households in buildings within a radius of 1mile/1.5 km from the accident site (household response 22.3%) and a control group of 292 residents (household response 23.3%) in buildings in Upper Manhattan, NY, more than 4.8 miles/9 km north of the accident site [3,4]. Participants who lived near the WTC had more upper and lower airway symptoms than the control subjects [3,4], but lung function was not significantly different (p > 0.05) [3]. Six years after a fire in a chemical waste depot containing batteries, acids, bases and paint and 480 metric tons of chemical waste was burnt in the town Drachten in the Netherlands, 138 subjects who had been present in the area at the time of the fire were telephone interviewed for lower respiratory symptoms as part of a case-control study [5]. The interviewed subjects with reported normal respiratory health before the accident were selected for pulmonary examination. All participants with new lower airway symptoms for more than three months after the fire were selected as cases (n = 25), a randomly selected group without these criteria (n = 99) was control. Cases were more likely to have been working or staying within 100 metres of the fire site (high exposed) than more than one kilometre away (low exposed) compared with control subjects. The cases had significantly lower forced expiratory volume one second per cent predicted (FEV_1_% predicted) and higher dose–response slope (DRS) with bronchial histamine provocation than control subjects. High exposed subjects had borderline significantly lower FEV_1_% predicted (p = 0.099) and borderline higher DRS with histamine provocation (p = 0.056) than low exposed subjects [5]. One to two years after the clean-up of the oil spill from the tanker Prestige off the coast of Spain, a questionnaire based cross-sectional study of 6869 costal fishermen (response rate 76%) from 38 cooperatives along the cost was performed. Local fishermen on the most polluted coastline who took part in clean-up activities had more airway symptoms than a control group of fishermen from non-polluted areas [6]. About two years after the accident a group of the most exposed fishermen (n = 501) and a group of fishermen from the low exposed costal line who did not take part in clean-up activities (n = 177) were examined for health effects from the exposure including lung function measurements [7]. This study confirmed the results from the questionnaire based study that exposed had significantly more airway symptoms than control subjects, but nasal symptoms and lung function was not significantly different between the exposure groups, neither was the non- specific bronchial reactivity measured with metacholine provocation [7].

These studies varied with respect to exposure, effect measures, time delayed between exposure and study and in study design. The population in the present study is relatively stable and homogenous, and together with the relatively low ambient air pollution expected to be present under normal circumstances in this area, this study could provide interesting information on the effect of an air polluting accident.

Another aspect of this kind of accident is the effect of prolonged media coverage on symptoms in the population. Individuals who have read and heard about disasters through the mass media may develop post- traumatic stress-related symptoms [8,9], and medically unexplained physical symptoms [9]. Adjusting for stress symptoms might lead to better estimates of event-related risk of self-reported symptoms in an affected population. However, possible persistent airway symptoms caused by air pollution from the accident may also contribute to longer lasting stress reaction related to the incident.

The main aim of this study was to assess the prevalence of airway symptoms and lung function in an adult population living close to an air-polluting explosion site compared with a population living far away.

## Methods

### Exposure

The tank that exploded contained remnants of sulphuric gasoline (coker gasoline), 50 m^3^ of alkalinised sludge containing sodium thiolates (mercaptans), 200 m^3^ of treated wastewater and 15 m^3^ of concentrated hydrochloric acid. The smoke content was not analysed, but thiols and other sulphides were probably oxidised, forming sulphuric dioxide (SO_2_) during the fire. Air measurements in the industrial harbour two to three weeks after the accident showed low levels of mercaptans [10]. Sulphuric compounds from the tanks were probably also spread to the soil and water in the industrial area.

Clean-up operations started soon after the accident. Contaminated soil was collected and stored in big bags, but was not removed from the harbour area until 2010, three years later.

In an area west, north and east of the accident site within a distance of six kilometres there are individual and small clusters of houses spread within the whole area mainly along the roads. This area is separated by fields, hills and woodland from houses further away. South and south west of the harbour is the fiord. Except for their neighbourhood to the industrial harbour the inhabitants in this area, which belong to two municipalities, live in a rural environment with low road traffic. The average distance from the accident site to the residential addresses for the inhabitants is about 3.7 kilometres (range 1–6 km).We anticipated that the highest environmental chemical exposure from the accident had been within this area, and all the inhabitants here were defined as exposed to the pollution from the accident. We also anticipated that people living 1–3 kilometres from the accident site had been more exposed to pollution from the accident than those living 3–6 kilometres away. The control group was selected from inhabitants living in two separate areas to the east and north east of the industrial harbour in the same two municipalities as the exposure area, but an average 28 kilometres away (range >20-30 km). We anticipated low accident related exposure in the control areas, but otherwise the residence environment was considered to be similar to the area where the exposed population lived, with small clusters of houses in a rural environment.

### Study design and population

This cross sectional study was carried out 18 months after the explosion, from November 2008 to March 2009. Information about residence was obtained from the National Population Register.

All inhabitants from 18 years old and upwards living within six kilometres (average distance 3.7 kilometres) of the accident site at the time of the incident were defined as exposed (N = 308). A random sample of inhabitants living more than 20 kilometres away (average distance 28 kilometres) in the same two municipalities, were defined as controls (N = 316). These controls had the same age and gender distributions as the exposed.

The population was informed about the study at a public meeting, through the local press, television channels and posters in public buildings. A letter inviting recipients to a health examination in the local municipality, a questionnaire and up to two reminders were sent by mail to each selected person.

Lists of workers employed in the industrial harbour at the time of the accident were obtained from the local employers. Inhabitants in the control group who worked in the industrial harbour were excluded from the study population.

A total of 402 individuals participated, 223 in the exposed group (115 men, response rate 70%, and 108 women, response rate 75%), and 179 in the control group (99 men, response rate 51% and 80 women, response rate 65%). In the exposed group, 59 men and 55 women lived within three kilometres, and 56 men and 53 women lived from three to six kilometres from the explosion site. The non-responder group comprised mostly of younger adults, especially younger men.

### Airway symptoms

Questions about symptoms from lower airways included (yes/no): Usually morning cough, daily cough, cough at least three months a year, cough with phlegm, cough with phlegm at least three months a year, dyspnoea while walking on flat ground, dyspnoea while walking uphill compared to others, ever had episodes of wheezing from chest, were taken from the ATS-DLD-78A questionnaire [11].

Questions about current symptoms from upper airways: Blocked nose, rhinorrhoea, irritated nose and sore throat, were taken from the Wasserfallen et al. validated questionnaire [12]. Participants were to report the degree of present symptoms on a five-point scale (0–4).

### Lung function

Spirometry was performed in accordance with the ATS/ERS standardisation recommendation from 2005 [13], but accepting 200 mL repeatability in accordance with the 1994 recommendation [14]. A dry wedge spirometer, ‘Vitalograph Gold Standard plus’ (model 2160) was used for all measurements. Among men, 99 spirograms were found to be acceptable in the exposed group and 91 in the control group; for women, the figures were 94 and 71, respectively.

The chosen forced expiratory volume in one second (FEV_1_) and forced vital capacity (FVC) were compared with reference (predicted) values from a non-smoking, healthy, west-coast Norwegian population [15]. Spirometry was performed before and 15 minutes after inhaling 0.4 mg adrenergic beta2-agonist salbutamol from a Discus inhalator [16]. Airway obstruction was defined as FEV_1_% predicted <80% and an FEV_1_/FVC ratio <0.70, which is moderate and severe obstruction according to the GOLD criteria [17].

### Post-traumatic stress symptoms

To assess stress symptoms relating to the 2007 accident, questions were included from the Impact of Event Scale- Revised (IES-R) [18]. This scale is suggested to be useful when screening for signs of post-traumatic stress (PTSD) [19]. It contains 22 questions on different stress symptoms experienced during the past week relating to the 2007 accident. Answers were given on a five-point scale, from 0 (not at all) to 4 (extreme). The maximum obtainable impact score was 88, a high number representing a high symptom level. A cut-off value of 22 was used as this has been shown to be optimal for classification accuracy, sensitivity and specificity of PTSD [20].

### Population characteristics and covariates

The participants were asked (yes /no): Have you had an infection in the preceding month, and have you cat or dog, moisture damage or carpeted floors in your home. They were also asked about years of education after lower secondary school, whether they were working, were on sick leave or rehabilitation, in receipt of disability pension, retirement pension or were students.

Subjects in employment were asked to state their occupation and industry using free text. Information about current occupation was coded as a three-digit code in accordance with the Norwegian version of the International Standard Classification of Occupation (ISCO −88) by two skilled researchers [21]. The individual’s occupation was then linked to a general population job-exposure matrix (JEM) which classifies each occupation into none, low or high exposure according to occupational exposure to mineral dust, biological dust or fumes/gases [22]. In the present study we classified these three exposure groups into two where the original none and low exposure groups were merged.

Information was also obtained about years of daily smoking, previous smoking (yes/no), and cigarettes per day for current smokers. Smoking was defined as present versus never/previous smoking.

A blood sample was taken by vein puncture, and the blood was coagulated in tubes at room temperature and centrifuged for serum within two hours. Serum was analysed at the laboratory at Haukeland University Hospital the next day using Phadiatop^®^ based on the Immuno-CAP-FEIA system (Phadia AB, Uppsala, Sweden). Subjects with positive Phadiatop^®^ (specific IgE toward one or more of the following airway allergens: *Dermatophagoides pteronyssinus, Cladosporium herbarum*, cat, dog, horse, birch, timothy and mugwort) results were defined as having atopy.

The height and weight of participants were measured while wearing indoor clothing without shoes and jacket. Height was used as a covariate.

The described population characteristics and covariates were assessed because they may be associated with respiratory health or lung function.

### Statistical analyses

Comparison of characteristics variables among exposed and controls were done by Pearson Chi-Square test, or Fisher exact test for small numbers in categorical variables. Continuous variables were compared by independent sample t-tests. However, when the continuous variables were not normally distributed, the Mann–Whitney U test was used.

The relationship between categorical airway symptoms and exposure groups was analysed by logistic regression, adjusting for smoking (present versus previous or never), present occupational exposure (high versus low), Phadiatop^®^ (positive versus negative), infection in the preceding month (yes versus no), age (continuous scale) and impact score (≥22 versus <22). The answers to the upper airway symptoms were dichotomised; answers 0 and 1 were defined as no symptom and 2 to 4 as symptom present. We categorized the answers to these questions, as the underlying variables are naturally categorical (like symptoms from lower airways) [23]. Regression analyses with adjustments were not performed if the number were less than six individuals in any of the exposure groups. The relationship between continuous values as spirometry results and exposure was analysed by linear regression models, adjusting for smoking, occupational exposure, Phadiatop^®^, infection in the preceding month, age and height.

Most comparisons were performed for both genders and for men and women separately. When analysing men and women together, adjustment for gender (women versus men) was done.

In order to investigate a possible exposure-response of air polluting airway effect on the population within the 6 kilometre distance from the site, we made post-hoc calculations by analysing the airway effects separately for those living within three kilometres and those living 3–6 kilometres of the site, both compared to the control group. In total, 59 men and 55 women lived within three kilometres (average distance 26 kilometres), and 56 men and 53 women lived from three to six kilometres from the explosion site (average distance 4.7 kilometres).

If there were missing data points such as if a participant did not fill in a question in the questionnaire, this question was not used for that person in analyses. An exception for omitting a missed data point was if it seemed logical not doing so out of the context for the question. If a person answered “no” to each of the questions “have you usually morning cough”, “daily cough” or cough with phlegm”, and did not answer the question “have you cough more than three months a year”, then we anticipated that the answer also should be “no” here, and we used the answer “no” in the analyse for that question.

The comparative tests were two-sided, and p-values below 0.05 were considered significant. SPSS 18.0 was used for statistical analyses.

The study was approved by the Regional Committee for Medical Ethics of Western Norway and Norwegian Social Science Data Services.

## Results

Most population characteristics were similar when the exposed group and control group were compared, including the possible non-occupational environmental risk factors for airway symptoms as cats and dogs at home, building moisture at home and floor carpets at home. The prevalence of atopy among men was significantly lower and present smoking among women significantly higher in the exposed group (Table 1). All respondents with an impact score ≥22 were in the exposed group except for one woman in the control group.

**Table 1:** Characteristics in men and women aged ≥18 years old according to exposure from oil tank explosion

		**Men**			**Women**		
		**Control n = 99**	**Exposed n = 115**	**P- value**	**Control n = 80**	**Exposed n = 108**	**P-value**
Age	AM (SD)^a^	48.3 (14.3)	48.2 (17.5)	0.95^b^	47.2 (14.9)	45.3 (15.9)	0.42^b^
At work	n (%)	81 (82)	84 (73)	0.13^c^	61 (76)	77 (71)	0.45^c^
Sick leave/rehabilitation	n (%)	0 (0)	3 (3)	-	7 (9)	8 (7)	0.74^c^
Disability pension	n (%)	5 (5)	8 (7)	0.78^c^	6 (8)	6 (6)	0.76^c^
Retirement pension	n (%)	9 (9)	18 (16)	0.15^c^	7 (9)	13 (12)	0.47^c^
Student	n (%)	5 (5)	3 (3)	0.48^c^	7 (9)	11 (10)	0.74^c^
Others	n (%)	0 (0)	0 (0)	-	0 (0)	2 (2)	-
Years of education after lower secondary school	Med (Q1,Q3)^d^	3 (1, 4.5)	3 (1, 4)	0.34^f^	3 (2, 6)	3 (2, 5)	0.45^f^
High occupational exposure	n (%)	37 (38)	37 (32)	0.39^c^	4 (5)	11 (10)	0.20^c^
Smoking status							
Present smoking	n (%)	25 (25)	34 (30)	0.54^c^	11 (14)	28 (26)	0.047^c^
Years	Med (Q1,Q3)^d^	22 (14, 30)	20 (10, 37)	0.98^f^	20 (20, 35)	22 (10, 30)	0.99^f^
Present cigarettes pr day	Med (Q1,Q3)^d^	10 (9)	12 (5)	0.37^f^	10 (9)	10 (5)	0.82^f^
Earlier smoked	n (%)	39 (39)	40 (35)	0.74^c^	30 (38)	31 (35)	0.56^c^
Years	Med (Q1,Q3)^d^	15 (10, 22)	18 (10, 30)	0.62^f^	15 (5, 20)	10 (8, 18)	0.71^f^
Never smoked	n (%)	33 (33)	41 (36)	0,84^c^	37 (46)	48 (44)	0,28^c^
Cats or dogs at home	n (%)	39 (39)	46 (40)	0.62^c^	34 (43)	50 (46)	0.61^c^
Building moisture at home	n (%)	8 (8)	8 (7)	0.90^c^	4 (5)	9 (8)	0.79^c^
Floor carpets at home	n (%)	3 (3)	2 (2)	0,66^e^	4 (5)	2 (2)	0.40^e^
Infection in the preceding month	n (%)	36 (37)	33 (29)	0.23^c^	25 (31)	39 (36)	0.46^c^
Impact score > =22	n (%)	0 (0)	11 (10)	-	1(1)	10 (9)	0.026^e^
Height (m)	AM (SD)^a^	1,79 (0,06)	1,79 (0,05)	0.71^b^	1,67 (0,05)	1,66 (0,06)	0.32^b^
Body mass index (kg/m^2^)	AM (SD)^a^	26.5 (3.7)	26.9 (4.0)	0.52^b^	27.6 (5.5)	26.3 (6.4)	0.14^b^
Phadiatop^®^ positive	n (%)	31 (31)	22 (19)	0.046^c^	15 (19)	25 (23)	0.45^c^

Exposed status was defined as living <6 km (average 3.7 km) from the explosion site; control status as living >20 km (average 28 km) from the explosion site

^a^ Arithmetic mean (Standard deviation). ^b^ Independent sample t-test. ^c^ Pearson Chi-Square test. ^d^ Med: Median. Q1: 25^th^ percentile. Q3: 75^th^ percentile

^e^ Fisher exact test. ^f^ Mann–Whitney U test.

Most airway symptoms were significantly more common in the group living less than six kilometres from the accident site than among control subjects (Table 2). The differences were also statistically significant for nose irritation, sore throat and cough in both men and women, adjusting for potential confounders (Table 3).

**Table 2:** Prevalence and odds ratio (OR) of airway symptoms according to exposure from oil tank explosion

		**Main group analysis**		**Sub-group analysis**				
	**Control**	**Exposed**	**Exposed (<6 km) vs control**	**Exposed 3–6 km**	**Exposed (3-6km) vs control**	**Exposed <3 km**	**Exposed (<3km) vs control**	**Linear trend:control/3-6 km/<3 km**
	n = 179	n = 223		n = 114		n = 109		
	n (%)	n (%)	OR (95% CI)^ab^	%	OR (95% CI)^ab^	%	OR (95% CI)^ab^	P-value^b^
Blocked nose	37 (21)	71 (32)	1.7 (1.0, 2.8)	28	1.5 (0.8, 2.6)	36	2.0 (1.1, 3.6)	0.020
Rhinorrhoea	24 (13)	52 (23)	1.9 (1.1, 3.4)	21	1.6 (0.9, 3.1)	26	2.4 (1.2, 4.6)	0.010
Irritated nose	24 (13)	79 (35)	3.4 (2.0, 5.9)	34	3.2 (1.7, 5.9)	37	3.7 (2.0, 7.0)	<0.001
Sore throat	22 (12)	73 (33)	3.1 (1.8, 5.5)	34	3.4 (1.8, 6.3)	31	2.9 (1.5, 6.6)	0.001
Morning cough	26 (15)	74 (33)	3.5 (2.0, 6.2)	33	3.0 (1.6, 5.7)	33	4.3 (2.2, 6.7)	<0.001
Daily cough	42 (24)	91 (42)	2.3 (1.4, 3.7)	38	1.8 (1.0, 3.1)	46	3.0 (1.7, 6.4)	<0.001
Cough > 3 mo/yr	21(12)	64 (29)	2.9 (1.5, 5.3)	24	2.1 (1.0, 4.3)	34	4.1 (2.0, 8.4)	<0.001
Cough with phlegm	45 (26)	82 (38)	1.9 (1.2, 3.1)	39	1.8 (1.0, 3.2	37	2.1 (1.2, 3.8)	0.010
Cough with phlegm > 3 mo/yr	11 (6)	39 (17)	2.9 (1.3, 6.2)	13	1.6 (0.6, 3.8)	22	5.7 (2.3, 14.2)	<0.001
Dyspnoeic walking flat	13 (7)	30 (14)	1.8 (0.9, 3.8)	15	1.8 (0.8, 4.0)	12	1.9 (0.8, 4.5)	0.132
Dyspnoeic walking uphill	44 (25)	75 (35)	1.5 (0.9, 2.4)	33	1.3 (0.7, 2.3)	37	1.7 (0.95, 3.0)	0.075
Ever chest wheeze	21 (12)	36 (16)	1.7 (0.8, 3.2)	15	1.3 (0.6, 2.9)	17	2.2 (0.98, 4.9)	0.061

Exposed status was defined as living <6 km (average 3.7 km) from the explosion site; control status as living >20 km (average 28 km) from the explosion site. Increasing intensity of exposure within the exposed group was defined by dividing the exposed group in those living 3–6 km (average 4.7 km) and <3 km (average 2.6 km) from the explosion site.

^a^ Odds ratio (95% confident interval). ^b^Logistic regression models with adjustment for smoking, occupational exposure, atopy (defined by Phadiatop^®^), infection in the preceding month, post-traumatic stress impact score, age and gender.

**Table 3:** Prevalence and odds ratio (OR) of airway symptoms according to exposure from oil tank explosion in men and women

		**Main group analysis**		**Sub-group analysis**				
	**Control**	**Exposed**	**Exposed (< 6km) vs control**	**Exposed 3–6 km**	**Exposed (3–6 km) vs control**	**Exposed <3 km**	**Exposed (<3 km) vs control**	**Linear trend: control/3-6 km/<3 km**
**Men**	n = 99	n = 115		n = 59		n = 56		
	n (%)	n (%)	OR (95% CI)^ab^	%	OR (95% CI)^ab^	%	OR (95% CI)^ab^	P-value^b^
Blocked nose	24 (24)	41 (36)	1.8 (0.9, 3.5)	32	1.5 (0.7,3.3)	39	2.2 (1.0, 5.0)	0.046
Rhinorrhoea	13 (13)	29 (25)	1.9 (0.8, 4.8)	24	2.2 (0.9,5.3)	27	2.8 (1.1, 7.3)	0.03
Irritated nose	15 (15)	45 (39)	3.1 (1.4, 7.2)	36	3.4 (1.5, 7.8)	43	4.9 (2.0, 12)	<0.001
Sore throat	15 (15)	37 (32)	2.5 (1.2, 5.2)	37	3.0 (1.3, 6.8)	27	1.8 (0.7, 4.7)	0.10
Morning cough	21 (21)	37 (32)	2.1 (1.0, 4.4)	29	1.4 (0.6, 3.3)	36	3.7 (1.5, 9.1)	0.007
Daily cough	30 (30)	52 (45)	2.0 (1.1, 3.7)	36	1.2 (0.6, 2.5)	56	3.7 (1.7, 8.2)	0.002
Cough > 3 mo/yr	16 (16)	36 (31)	2.5 (1.2, 5.3)	24	1.5 (0.6, 3.7)	39	4.6 (1.8, 11)	0.008
Cough with phlegm	31 (32)	50 (44)	1.9 (1.0, 3.6)	45	1.6 (0.7, 3.3)	45	2.4 (1.1, 5.2)	0.03
Cough with phlegm > 3 mo/yr	9 (9)	25 (22)	2.7 (1.1, 6.6)	14	1.2 (0.4, 3.6)	30	6.7 (2.3, 20)	0.003
Dyspnoeic walking flat	9 (9)	13 (12)	1.1 (0.4, 3.0)	16	1.4 (0.5, 4.1)	7	0.7 (0.2, 3.0)	0.81
Dyspnoeic walking uphill	20 (21)	38 (33)	1.8 (0.9, 3.7)	36	1.7 (0.7, 3.7)	33	2.1 (0.9, 5.0)	0.08
Ever chest wheeze	13 (13)	19 (17)	1.7 (0.7, 4.5)	18	1.5 (0.5, 4.5)	16	2.1 (0.6, 6.7)	0.23
**Women**	n = 80	n = 108		n = 55		n = 53		
Blocked nose	13 (16)	30 (28)	1.6 (0.7, 3.4)	24	1.4 (0.6, 3.6)	32	1.7 (0.7, 4.2)	0.25
Rhinorrhoea	11 (14)	23 (22)	1.5 (0.7, 3.5)	18	1.2 (0.4, 3.1)	25	2.0 (0.8, 5.2)	0.17
Irritated nose	9 (11)	34 (32)	2.9 (1.2, 6.7)	33	3.0 (1.1, 7.6)	30	2.8 (1.0, 7.5)	0.04
Sore throat	7 (9)	36 (33)	4.1 (1.6, 10)	31	3.7 (1.3, 10)	36	4.7 (1.6, 13.7)	0.003
Morning cough	5 (6)	37 (34)	-	38	-	30	-	-
Daily cough	12 (15)	39 (36)	2.5 (1.1, 5.5)	39	2.6 (1.1, 6.5)	36	2.4 (0.95, 6.0)	0.054
Cough > 3 mo/yr	5 (6)	28 (26)	-	24	-	28	-	-
Cough with phlegm	14 (19)	32 (30)	2.0 (0.9, 4.6)	33	2.0 (0.8, 4.8)	28	2.2 (0.8, 4.9)	0.09
Cough with phlegm > 3 mo/yr	2 (3)	14 (13)	-	13	-	13	-	-
Dyspnoeic walking flat	4 (5)	17 (16)	-	15	-	17	-	-
Dyspnoeic walking uphill	24 (30)	37 (34)	1.0 (0.5, 2.0)	29	0.8 (0.4, 1.9)	42	1.3 (0.6, 2.8)	0.63
Ever chest wheeze	8 (10)	17 (16)	1.4 (0.5, 3.8)	13	1.0 (0.3, 3.4)	19	1.9 (0.6, 6.0)	0.29

Exposed status was defined as living <6 km (average 3.7 km) from the explosion site; control status as living >20 km (average 28 km) from the explosion site. Increasing intensity of exposure within the exposed group was defined by dividing the exposed group in those living 3–6 km (average 4.7 km) and <3 km (average 2.6 km) from the explosion site.

^a^ Odds ratio (95% confidence interval). ^b^Logistic regression models with adjustment for smoking, occupational exposure, atopy (defined by Phadiatop^®^), infection in the preceding month, post-traumatic stress impact score, age.

Those in the exposed group living less than three kilometres from the accident site had higher odds ratios for most symptoms compared to those living three to six kilometres away. This trend was significant for blocked nose, rhinorrhoea, nose irritations and cough among men (Table 3), and in addition for sore throat when both genders were analysed together (Table 2). The exposure-response pattern appeared to be less consistent among women than among men (Table 3).

The significant odds ratios in Tables 2 and 3 were still significant after sensitivity testing by excluding participants with impact score above 21 (data not given). In the exposed group living within six kilometres of the accident site, 42 individuals (32 men and 10 women) worked in companies at the site of the accident in May 2007. Adding an adjustment for work in these companies to the regression model did not change the results in Tables 2 and 3 (data not given).

Analysing the relationship between daily coughing and exposure group (home address) yielded a significant relationship between the symptom and residence less than three kilometres from the accident site (Table 4). Smoking, Phadiatop^®^ and impact score also had odds ratios above two, but among men estimates were higher for home address close to the accident site (Table 4).

**Table 4:** Logistic regression models for daily cough among men and women after the oil tank explosion

		**Daily cough**	
		**Men**	**Women**
		**OR (95% CI)**^**a**^	**OR (95% CI)**^**a**^
Distance between home address and explosion site	>20 km	1.0	1.0
	3-6 km	1.2 (0.6, 2.5)	2.6 (1.1, 6.5)
	<3 km	3.7 (1.7, 8.2)	2.4 (1.0, 6.0)
Smoking	Present vs ex/never	2.9 (1.4, 5.7)	3.5 (1.5, 8.0)
Occupational exposure	High vs low	1.2 (0.6, 2.3)	1.3 (0.4, 4.8)
Atopy	Phadiatop^®^ pos vs neg	2.3 (1.1, 5.0)	2.6 (1.1, 3.6)
Infection in the preceding month	Yes vs no	1.8 (0.9, 3.4)	1.7 (0.8, 3.6)
Impact score	≥22 vs <22	3.5 (0.8, 6.1)	2.1 (0.5, 8.9)
Age	Continuous	1.03 (1.00, 1.05)	1.00 (0.98, 1.03)

^a^ Odds ratio (95% confidence interval).

Linear multiple regression analyses with men and women together and controlling for covariates, showed lower lung function in the exposed group living <6 km from the accident site than in the control group living >20 km away, significant for FEV_1_ (mL), FVC (mL) and FVC% predicted (Table 5). In linear multiple regression analyses lower FEV_1_ (mL) and FVC (mL) were also found among both men and women in the exposed group (<6km) when controlling for covariates, but the findings were only statistically significant for FVC among men (Table 6). Among men, these lung function values were lower in the within-three-kilometre group than the three-to-six-kilometre group (Table 6).

**Table 5:** Lung function measures according to exposure from oil tank explosion

	**Control**	**Exposed**	**Difference between exposed and control**
	**n = 162**	**n = 193**	
	AM (SD)^a^	AM (SD)^a^	AM difference ^a^ (95% CI)^b^
FEV_1_ (mL)	3437 (860)	3273 (915)	−123 (−232, -14)
FEV_1_% predicted	92.9 (13.6)	89.5 (15.1)	−2.5 (−5.5, 0.5)
FVC (mL)	4486 (1046)	4248 (1076)	−173 (−297, -50)
FVC % predicted	99.7 (11.9)	96.3 (13.4)	−3.1 (−5.9, -0.4)
FEV_1_/FVC ratio	0.767 (0.072)	0.766 (0.083)	−0.001 (0.014, 0.013)
	n (%)	n (%)	Odds ratio (95% CI)^d^
Airway obstruction^c^ before salbutamol	8 (5)	21 (11)	2.5 (1.0, 6.2)
Airway obstruction^c^ after salbutamol	5 (3)	15 (8)	3.1 (0.98, 9.6)

Exposed status was defined as living <6 km (average 3.7 km) from the explosion site; control status as living >20 km (average 28 km) from the explosion site

^a^ Arithmetic mean (Standard deviation). ^b^ Arithmetic mean difference (95% confidence interval) between exposed group and control group from linear regression models adjusted for smoking, occupational exposure, atopy (defined by Phadiatop^®^), infection in the preceding month, height, age and gender (models for FEV1% predicted and FVC% predicted not adjusted for height, age and gender). ^c^ Airway obstruction defined as FEV_1_/FVC < 0.70 and FEV_1_% predicted <0.80. ^d^ Odds ratio (95% confidence interval) between exposed group and control group from logistic regression models with adjustment for smoking, occupational exposure, atopy (defined by Phadiatop^®^), infection in the preceding month, age and gender.

**Table 6:** Lung function measures according to exposure from oil tank explosion in men and women

	**Control**	**Exposed**	**Difference between exposed <6 km and control**	**Difference between exposed 3-6km and control**	**Difference between exposed <3 km and control**
**Men**	n = 91	n = 99			
	AM (SD)^a^	AM (SD)^a^	AM difference (95% CI)^b^	AM difference (95% CI)^b^	AM difference (95% CI)^b^
FEV_1_ (mL)	3860 (827)	3746 (843)	- 137 (−307, 32)	−87 (−336, 161)	−194 (−406, 18)
FEV_1_% predicted	90.7 (13.8)	88.5 (14.9)	−1.6 (−5.7, 2.5)	−0.6 (−6.6, 5.4)	−2.7 (−8.8, 3.5)
FVC (mL)	5125 (843)	4902 (880)	−242 (−435, -50)	−212 (−497, 73)	−275 (−569, 18)
FVC% predicted	99.5 (11.8)	96.1 (13.0)	−3.7 (−7.4, 0.0)	−2.4 (−7.9, 3)	−5.1 (−10.6, 0.5)
FEV_1_/FVC ratio	0.75 (0,08)	0.76 (0,08)	0.01 (−0.01, 0.03)	0.02 (−0.01, 0.05)	0.00 (−0.02, 0.03)
	n (%)	n (%)			
Airway obstruction ^c^ before salbutamol	5 (6)	14 (14)			
Airway obstruction ^c^ after salbutamol	4 (4)	9 (9)			
**Women**	n = 71	n = 94			
	AM (SD)^a^	AM (SD)^a^	AM difference (95% CI)^b^	AM difference (95% CI)^b^	AM difference (95% CI)^b^
FEV_1_ (mL)	2896 (543)	2775 (700)	−99 (−235, 37)	−206 (−402, -10)	5 (−190, 199)
FEV_1_% predicted	95.7 (12.8)	90.6 (15,4)	−3.5 (−8.1, 1.1)	−7.2 (−13.9, -0.5)	0.0 (−6.6, 6.5)
FVC (mL)	3667 (625)	3567 (807)	−92(−250, 87)	−252 (−478, -27)	66 (−158, 289)
FVC% predicted	99.8 (12,2)	96.6 (13.8))	−1.6 (−6.4, 3.2)	−6.5 (−12.6, -0.4)	1.0 (−5.0, 7.0)
FEV_1_/FVC ratio	0.80 (0.06)	0.77 (0.09)	−0.01 (−0.3, 0.01)	−0.01 (−4.2, 2.3)	−1.4 (−4.6, 1.9)
	n (%)	n (%)			
Airway obstructionc before salbutamol	3 (4)	7 (7)			
Airway obstructionc after salbutamol	1 (2)	6 (6)			

Exposed status was defined as living <6 km (average 3.7 km) from the explosion site; control status as living >20 km (average 28 km) from the explosion site. Increasing intensity of exposure was defined by dividing the exposed in those living 3–6 km (average 4.7 km) and <3 km (average 2.6 km) from the explosion site

^a^ Arithmetic mean (Standard deviation). ^b^ Arithmetic mean difference (95% confidence interval) between exposure groups and control group from linear regression models adjusted for smoking, occupational exposure, atopy (defined by Phadiatop^®^ ), infection in the preceding month, height and age (models for FEV_1_% predicted and FVC% predicted not adjusted for height and age). ^c^ Airway obstruction defined as FEV_1_/FVC < 0.70 and FEV_1_% predicted <0.8.

In a logistic regression model adjusting for covariates there were more obstructive lung functions, defined as FEV_1_% predicted below 80% and an FEV_1_/FVC ratio below 0.70, in the exposed group for men and women together, and were significant for pre-bronchodilator measurements and nearly significant (p = 0.054) for post-bronchodilator measurements compared to the control group (Table 5). The prevalence of obstructive lung function was also higher in the exposed group for men and women separately (Table 6), and the figures became significant when analysing the genders together. The association of exposure with more symptoms and lower lung function was apparently similar among atopic and non-atopic (defined by Phadiatop^®^) in stratified analyses (Additional file 1.Table 1 in on-line repository and Table 2 in on-line repository).

## Discussion

One and a half years after an explosion and fire in two tanks in a small industrial harbour, the individuals living within six kilometres of the explosion site had a doubled risk of airway symptoms as compared to a control group living more than twenty kilometres away. This was found for both upper and lower airway symptoms. An exposure-response relationship was significant with more symptoms among individuals living within three kilometres from the explosion site as compared to those living slightly further away, and this trend was also significant for men separately. The exposed individuals also had lower lung function and more airway obstruction (GOLD II/III) compared with the control group. Furthermore lung function was lower both among exposed men and women separately and became significant when analysing the genders together.

The findings of more airway symptoms in the exposed group are in accordance with a study conducted eight to 16 months after the WTC incident, where residents living within one and a half kilometres from ground zero had more coughs, wheezes and dyspnoea [3], and more nose irritation and sore throats [4], than a control group of residents living more than nine kilometres away. Similarly, there was a strong association between exposure to combustion products within 100 metres of the Drachten fire and persistent coughing, wheezing and dyspnoea six years later [5]. This study differed from ours in terms of the distance from the pollution site and time since exposure. In a questionnaire-based interview study carried out one to two weeks after the oil spill caused by the tanker Braer running aground in Shetland, people living within four and a half kilometres from the wreck reported more sore throats, itchy eyes, skin irritation and headaches than a control population living 95 kilometres away [24]. Symptoms diminished significantly in the exposed group over time, but throat irritation and breathlessness on exertion were still higher than in the control group after six months [25].

In our study, there were significantly more airway symptoms in the exposure group, also when adjusting for the PTSD-related impact scores. Individuals living within four and a half kilometres from the wreck of the Braer had more depressive and PTSD-related symptoms than the control group six months after the accident [25]. Three months after the WTC disaster, workers employed by a company near ground zero reported more symptoms, including coughing, wheezing, eye irritation and nose/throat irritation, but also more depressive and PTSD symptoms than office workers in the same company in Dallas [2]. In analyses of the Prestige oil spill study, a significantly higher risk of lower respiratory tract symptoms was found among fishermen who took part in clean-up activities, after adjustments were made for reported anxiety and participants believing that the exposure had affected their health [6]. The same adjustments were made in the survey of residents seven weeks after the grounding of the oil tanker Sea Empress, where residents showed more symptoms, including runny noses, sore throats and coughs than a control group [26].

The exposed participants in our study had significantly lower lung function and more spirometric airway obstruction. This has not been found or analysed in previous studies of lung function after similar accidents. When comparing exposed and control groups, lung function was not significantly different in connection with the Drachten fire [5], among residents living around ground zero, NY [3], and in the Prestige oil spill studies [7].

As this was a cross-sectional study and the population was not examined before the explosion, it is difficult to know whether the higher frequency of airway symptoms, lower lung function and signs of airway obstruction in the exposed group were caused by pollution from the oil tank explosion or by other factors. The contamination from the explosion consisted of different hydrocarbon compounds mixed with sulphur products, and the pollution in the area lasted for two years. However, information about concentration levels of air pollutants in the area was scarce, and the exposure level was probably low. Previous studies have shown bronchial effects of sulphur dioxide [27,28], and people exposed to malodorous sulphuric air pollution including hydrogen sulphide and mercaptans as a result of living near pulp mills reported more coughing, pharyngeal irritation, breathing problems and eye symptoms than control groups living in a non-polluted community [29]. Thus, it seems plausible that the findings could be related to air pollution. However, there may have been air pollution from other sources before the event, such as from the ordinary industrial activity in the industrial harbour. This cannot be assessed in the present study, as we have no information about the health of the population before the explosion. This study took part in two rural municipalities with no other industrial emission sites than the industrial harbour, with generally low road traffic and without town or city in the neighbourhood.

Strengths of our study are that both exposed and control individuals were recruited from the same municipalities, and that the groups were comparable except for their relationship to the accident site. The variables indicating socioeconomic and demographic conditions confirmed that the groups were highly comparable. The few factors that were different were adjusted for in the analyses. We used present smoking as a factor for the effect of smoking on the airways. Pack years might have been a better measure, but we did not have this information. However, years of smoking and present cigarettes smoked per day among smokers were not significantly different between the groups.

The low response rate in the control group may have resulted in selection bias, but we do not know whether this could have influenced the two groups differentially. The majority of the non-responders were younger people, especially younger men. In the examined municipalities, some individuals stay away from their homes for days at a time because of working or studying elsewhere. They could have been among the healthier non-responders and thus have contributed to lower odds ratio in the study than expected in the total population. Some anticipate more health problem among non-responders [30]. If so, we would have expected lower odds ratios than registered in this study.

## Conclusions

Individuals who lived near the industrial harbour where two tanks with a heterogeneous mixture of sulphuric hydrocarbons exploded and caught fire in May 2007 had more upper and lower airway symptoms, lower lung function and more airway obstruction than a comparable control group one and a half years after the accident. Both pollution from the accident and the industrial activities in the industrial harbour may have contributed. A follow-up study of the population in question will be performed to investigate long-term consequences of the accident. The findings add to reports on how industrial accidents may have health consequences for the general population living near an industrial plant in an otherwise rural area. Industrial planning must include the health concern of the general population also in case of accidents.

## Abbreviations

FVC: Forced vital capacity; FEV_1_: %predicted Forced expiratory volume in one second; IES-R: Impact of Event Scale- Revised; GOLD: The Global Initiative for Chronic Obstructive Lung Disease.

## Competing interests

All authors declared that they have no competing interest.

## Authors’ contributions

JTG contributed in designing and data collection, undertook the analyses, drafted and revised the manuscript after consultation with the other authors. MB, BEM, ÅI, BEH; NM contributed to the study design and revised and approved the manuscript. BEH and NM took part in data collection and ÅI in statistical analyses. CS revised and approved the manuscript. All authors read and approved the final manuscript.

## Pre-publication history

The pre-publication history for this paper can be accessed here:

http://www.biomedcentral.com/1471-2466/12/76/prepub

## Supplementary Material

Additional file 1 **Airway symptoms and lung function measures according to oil tank explosion among Phadiatop^®^ positive and negative for exposed related to controls.** Table 1 in on-line repository. Prevalence of airway symptoms among exposed and control subjects ≥18 years old stratified on Phadiatop^®^ negative and positive, and odds ratios for exposed with controls as reference. Table 2 in on-line repository. Spirometry results among exposed and control subjects ≥18 years old stratified on atopy status; Phadiatop^®^ negative and positive, and adjusted mean difference between exposed and controls. Click here for file
