# Supplementary material for: Airway symptoms and lung function in the local population after the oil tank explosion in Gulen, Norway
Source: BMC Pulm Med. 2012 Dec 12;12:76. doi: 10.1186/1471-2466-12-76 (PMC3549740; doi:10.1186/1471-2466-12-76)
Supplement: Additional file 1 — Airway symptoms and lung function measures according to oil tank explosion among Phadiatop® positive and negative for exposed related to controls. Table 1 in on-line repository. Prevalence of airway symptoms among exposed and control subjects ≥18 years old stratified on Phadiatop® negative and positive, and odds ratios for exposed with controls as reference. Table 2 in on-line repository. Spirometry results among exposed and control subjects ≥18 years old stratified on atopy status; Phadiatop® negative and positive, and adjusted mean difference between exposed and controls. [file 1471-2466-12-76-S1.docx]

Table 1 in on-line repository. Prevalence of airway symptoms among exposed and control subjects ≥18 years old stratified on Phadiatop® negative and positive, and odds ratios for exposed with controls as reference. Exposed: living <6 km from the accident site in May 2007. Controls: living >20 km from the explosion site.

|  | Phadiatop® negative | | |  | Phadiatop® positive | | |
| --- | --- | --- | --- | --- | --- | --- | --- |
|  | Control | Exposed | Exposed vs control |  | Control | Exposed | Exposed vs control |
|  | n (%) | n (%) | OR (95% CI)^a^ |  | % | % | OR (95% CI)^a^ |
|  | n=133 | n=174 |  |  | n=46 | n=47 |  |
|  |  |  |  |  |  |  |  |
| Blocked nose | 24 (18) | 48 (28) | 1.60 (0.90, 2.85) |  | 13 (28) | 22 (47) | 1.96 (0.71, 5.44) |
| Rhinorrhoea | 17 (13) | 37 (21) | 1.78 (0.93, 3.43) |  | 7 (15) | 15 (32) | 2.95 (0.84, 10.4) |
| Irritated nose | 16 (12) | 58 (33) | 3.36 (1.79, 6.33) |  | 8 (17) | 20 (43) | 3.80 (1.14, 12.7) |
| Sore throat | 16 (12) | 58 (33) | 3.03 (1.58, 5.82) |  | 6 (13) | 19 (40) | 3.39 (1.05, 11.0) |
| Morning cough | 15 (11) | 53 (31) | 3.63 (1.82, 7.24) |  | 11 (24) | 21 (45) | 3.47 (1.15, 10.5) |
| Daily cough | 26 (20) | 65 (38) | 2.18 (1.25, 3.80) |  | 16 (37) | 26 (58) | 2.49 (0.85, 7.28) |
| Cough>3 mo/yr | 11 (8) | 47 (27) | 3.55 (1.64, 7.72) |  | 10 (30) | 17 (42) | 1.44 (0.47, 4.48) |
| Cough with phlegm | 30 (24) | 57 (34) | 1.75 (1.00, 3.06) |  | 15 (34) | 25 (54) | 3.03 (1.01, 9.09) |
| Cough with phlegm>3 mo/yr | 8 (6) | 29 (17) | 2.85 (1.16, 6.99) |  | 3 (10) | 10 (27) | 2.34 (0.45, 12.2) |
| Dyspnoeic walking flat | 7 (5) | 23 (14) | 2.79 (1.12, 6.96) |  | 6 (13) | 7 (15) | 0.60 (0.15, 2.41) |
| Dyspnoeic walking uphill | 32 (25) | 53 (32) | 1.42 (0.82, 2.48) |  | 12 (26) | 21 (46) | 1.35 (0.50, 3.67) |
| Ever chest wheeze | 8 (6) | 23 (13) | 2.52 (0.99, 6.42) |  | 13 (28) | 13 (28) | 0.80 (0.27, 2.36) |

^a^ Odds ratio (95% confidence interval) between exposed and control group from logistic regression models adjusted for smoking (present (1) vs never/ex(0)), occupational exposure (high vs low), infection in the preceding month (yes (1) vs no (0)), age (continuous scale), impact score (>=22 (1) vs <22 (0)), gender (women (1) vs men (0)).

Table 2 in on-line repository. Spirometry results among exposed and control subjects aged ≥18 years old stratified on atopy status; Phadiatop® negative and positive, and adjusted arithmetic mean difference between exposed and controls. Exposed group: living < 6 km from the accident site in May 2007. Control group: living >20 km from the explosion site.

|  |  |  | | |  | | |
| --- | --- | --- | --- | --- | --- | --- | --- |
|  |  | Phadiatop® negative | | | Phadiatop® positive | | |
|  |  | Control | Exposed | Difference between exposed and control | Control | Exposed | Difference between exposed and control |
|  |  | AM (SD)^a^ | AM (SD)^a^ | AM difference (95% CI)^b^ | AM (SD)^a^ | AM (SD)^a^ | AM difference (95% CI)^b^ |
|  |  | n=122 | n=148 |  | n=40 | n=43 |  |
| FEV_1_ (mL) |  | 3376 (825) | 3264 (928) | -104 (-230, 23) | 3625 (945) | 3296 (898) | -244 (-469, -209) |
| FEV_1_% predicted |  | 93.2 (13.6) | 90.0 (15.6) | -2.4 (-5.9, 1.1) | 91.9 (13.6) | 88.1 (13.8) | -2.4 (-8.1, 3.4) |
| FVC (mL) |  | 4410 (1007) | 4254 (1072) | -142 (-280, -4) | 4717 (1139) | 4201 (1119) | -345 (-643, -47) |
| FVC % predicted |  | 99.8 (11.4) | 97.0 (13.3) | -2.7 (-5.7, 0.4) | 99.1 (13.6) | 93.5 (13.6) | -4.4 (-10.6, 1.7) |
| FEV_1_/FVC ratio |  | 0.766 (0.0745 | 0.763 (0.086) | -0.004 (-0.020, 0.012) | 0.768 (0.067) | 0.779 (0.073) | 0.006 (-0.025, 0.037) |
|  |  |  |  |  |  |  |  |

^a^ Arithmetic mean (Standard deviation). ^b^ Arithmetic mean difference (95% confidence interval) between exposed (living <6 km) and control group (living >20 km) from linear regression models adjusted for smoking (present (1) vs ex/never (0)), occupational exposure (high (1) vs low/none (0)), infection in the preceding month (yes (1) vs no (0)), height (continuous scale), age (continuous scale) and gender (women (1) vs men (0) (models for FEV1 % predicted and FVC % predicted not adjusted for height, age and gender).
